# Microbial Production of Short Chain Fatty Acids from Lignocellulosic Biomass: Current Processes and Market

**DOI:** 10.1155/2016/8469357

**Published:** 2016-07-31

**Authors:** Ivan Baumann, Peter Westermann

**Affiliations:** Department of Sustainable Biotechnology, Aalborg University, Copenhagen, A.C. Meyers Vaenge 15, 2450 Copenhagen SV, Denmark

## Abstract

Biological production of organic acids from conversion of biomass derivatives has received increased attention among scientists and engineers and in business because of the attractive properties such as renewability, sustainability, degradability, and versatility. The aim of the present review is to summarize recent research and development of short chain fatty acids production by anaerobic fermentation of nonfood biomass and to evaluate the status and outlook for a sustainable industrial production of such biochemicals. Volatile fatty acids (VFAs) such as acetic acid, propionic acid, and butyric acid have many industrial applications and are currently of global economic interest. The focus is mainly on the utilization of pretreated lignocellulosic plant biomass as substrate (the carbohydrate route) and development of the bacteria and processes that lead to a high and economically feasible production of VFA. The current and developing market for VFA is analyzed focusing on production, prices, and forecasts along with a presentation of the biotechnology companies operating in the market for sustainable biochemicals. Finally, perspectives on taking sustainable product of biochemicals from promise to market introduction are reviewed.

## 1. Introduction

In 1996, the report “Technology vision 2020” [1] was published by the US chemical industry. The work was promoted by the White House Office of Science and Technology Policy and focused on future needs in research and development (R&D). But the report only pinpointed market issues as the important challenges for the chemical industry towards 2020. Three years later, a second report was published [2] with a completely different content and perspective for the chemical industry: New Biocatalysts, Essential Tools for a Sustainable 21st Century Chemical Industry. The report promoted government initiatives and included the extensive studies that had been carried out by the Pacific Northwest National Laboratory (PNNL), in collaboration with the National Renewable Energy Laboratory (NREL), and by the Office of Biomass Program. In 2004, their first report was published, entitled Top Value Added Chemicals From Biomass [3]. The ultimate task was “…to identify the top ten opportunities for the production of value-added chemicals from biomass that would economically and technically support the production of fuels and power in an integrated bio-refinery and identify the common challenges and barriers of associated production technologies.”

Initially, the authors developed a catalogue with a list of more than three hundred putative building block molecules, all having the potential for biocatalytic production from biomass. The list was then narrowed down to end up with almost fifty potential building block candidates. Among those were VFAs such as acetic acid and propionic acid. Acetic acid was chosen as commodity chemical and as a reagent adding functionality to hydrocarbons by supplementing with two carbon units. Propionic acid was selected as a reagent and a building block compound.

Ultimately, the authors identified and listed fifteen chemicals that could be produced from carbohydrates and suggested the compounds as targets for intensified scientific research. The choice of targets was based upon factors such as established conversion processes, the ability of a compound to serve as a platform for the production of derivative compounds, industrial viability, and economic aspects such as market size [4]. Although VFAs were not included among the ultimate top fifteen compounds, the 2004 report resulted in an increased research in sustainable organic acid production [5].

VFAs are widely used building block chemicals, which are employed in the manufacturing of a wide range of chemicals, pharmaceuticals, and materials, or they are used as free acids for, for example, feed conservation in the agricultural industry.

Acetic acid and derivatives are applied in a range of industries such as the electronic industry, polymer industry, chemical industry, and the food industry. The acid has many uses including the following: as an etching agent [6], as a component in detergents used for manufacturing of microelectronics; in the production of lignin-containing polyurethane [7], as a component in the manufacturing of hydrophobic and lipophobic papers in the polymer industry [8], in polyethylene production, and as an important preservation ingredient in the food industry. The acid is the principal compound in vinegar [9] and is used in numerous industrial and household food products and preparations [10].

Propionic acid and its derivatives are used either directly or as building block chemicals in a range of industries and in agriculture. Propionic acid is used without modification as a preservative in the food industry. The acid and its salts, such as sodium and calcium propionate, are used in agriculture for animal feed and grain preservation [11, 12]. A recent study on feed preservation demonstrated that treatment with a combination of acetic acid and propionic acid conserved well and prolonged storage in comparison to a nontreated control [13]. Moreover, propionic acid is used directly or as a modified compound in the manufacture of herbicides [14], as building block in pharmaceuticals [15] and in cellulose acetate propionate (CAP) plastics in the polymer industry [16].

Swedish company Perstorp Speciality Chemicals AB is marketing a product consisting of propionic acid and propionic acid glycerol esters. The mixture is an animal feed preservative that inhibits growth of molds and yeasts at a dosage of around 0.5–2.7% in stored grains [17].

Butyric acid and its derivatives have broad applications within the food industry, perfume and fragrance industries, the polymer industry, and the pharmaceutical industry.

Food and beverage industries use butyric acid directly to add or enhance a butter-like taste in food and beverages. Esters of butyric acid are generally aromatic and due to their fruity fragrance, they are used as additives for increasing fruit fragrance and as aromatic compounds in the production of perfumes [18]. Butyric acid is also used to synthesize butyryl polymers such as cellulose acetate butyrate (CAB) in the polymer industry [19]. In human health applications, butyric acid is a component of prodrugs with demonstrated anticancer effects [20]. Furthermore, derivatives of butyric acid from endogenous bacteria are known to promote colon health, and the acid presumably also has therapeutic clinical effects [21]. Currently, most VFAs used in industry are produced by petrocatalysis from refined heavy oil and natural gas. Naphtha and syngas are examples of such refined products, which might be further refined and processed by organic catalysis and synthesis to VFAs [22] that in turn serve as platforms for further organic synthesis based upon reaction with the terminal carboxylic group of the acids. Petrocatalysis involves high pressure and temperature process conditions requiring high energy inputs [23] and about 4% of global oil consumption is currently related to the production of chemicals and plastics [24]. Although this is a relatively low figure, VFA production from oil creates hazardous wastes such as heavy metals and organic solvents in addition to the emission of greenhouse gasses.

Acetic acid is produced mainly from mineral oil and natural gas either through methanol carbonylation or acetaldehyde oxidation [22].

The primary route of propionic acid synthesis employs the Oxo-synthesis process by hydrocarboxylation of ethylene in the presence of a nickel carbonyl catalyst or a rhodium catalyst but liquid-phase oxidation of propionaldehyde also yields propionic acid [22].

Butyric acid can be prepared chemically by oxidation of butyraldehyde obtained from propylene by Oxo-synthesis similar to propionic acid synthesis [25].

However, oil-derived chemicals can be produced from biomass instead [26], because, for example, lignocellulosic biomass has a chemical composition similar to fossil feedstocks and pretreated lignocellulosic biomass offers chemical compounds comprising different functional groups that facilitate chemical processing [27].

VFA can also be synthesized by microbial fermentation that requires three distinct process units: (1) biomass conversion, (2) fermentation, and (3) the recovery of the products from the fermentation broth [28, 29]. Even though petrocatalysis has been used for many years, there might be reasons to shift to biological processes instead. However, the sustainability of microbial conversion is still of major concern.

## 2. Sustainability of Biomass-to-Chemicals Process

If biomass-to-chemicals processes employ renewable feedstocks in integrated unit operations with recycling and exhaustive use of raw materials and energy, then they meet a rough definition of a biorefinery.

To become specific, biomass feedstocks must have important potential advantages over fossils. For example, carbon emitted to the atmosphere from conversion of renewable biological materials has a net zero carbon impact on the atmosphere's chemical composition [30, 31]. In other words the carbon is part of a closed loop whereby plant growth recaptures the carbon that is emitted during biomass use and its conversion. In addition to this, plant biomass is usually a domestic resource, which can be obtained at little cost [32]. Indeed, one requirement is that the biomass is of second-generation origin and has been grown and harvested without upsetting food supplies and supplies of feed and fiber [33]. Moreover, biomass conversion to organic chemicals such as VFA requires several chemical reactions in succession, which in turn require energy. Therefore, carbon neutrality can only be achieved if the energy, that is used to power the processes, is from renewable sources.

Although there are exceptions to the notion that biomass use is carbon neutral, especially within the context of biofuels and bioethanol [34], the notion is rarely challenged [35]. But even biomass from dedicated crops might not be carbon neutral. On the contrary, agriculture can potentially increase atmospheric CO_2_ because emissions are highly dependent on where and how the biomass is grown and harvested [31, 35, 36].

A thorough discussion of carbon footprint and putative carbon neutrality in the production of biochemicals is beyond the frame of this review. What should be mentioned though is the difference to an overall carbon footprint between direct combustion of lignocellulosic biomass and the carbon capture by incorporation of carbon into chemicals. To some extent, the latter alternative makes up a contemporary carbon sink.

Interest in the biomass-to-chemicals value chain has increased sharply during the recent ten years within industrial companies. Such interest has accelerated R&D into development of sustainable biomass conversion-to-chemicals processes [37] and there are specific factors on the production side in favor of sustainably produced chemicals to substitute for petrochemical counterparts. When chemicals are produced from biomass, the biorefinery saves energy and mitigates CO_2_ emissions [38] and it has been put forward that industrial biorefineries, as opposed to oil refineries, often show higher reaction rates, increased conversion efficiencies, improved product purities, and reduced chemical waste generation [39]. On the demand side, consumers wish for “natural” and “green” preservatives, fragrances, and materials [40]. In some markets, consumers prefer food additives or pharmaceutical products containing ingredients of natural origin. They are considered “healthier” and customers are often ready to pay more for such natural products. Moreover, biomass-derived chemicals are considered safer for human health than oil-derived products [41, 42].

A frequent argument put forward in favor of transition away from petrochemistry toward biochemistry is the rising oil prices and the finite nature of oil reserves. According to the International Energy Outlook 2013 [43], global energy consumption will grow by 56 percent between 2010 and 2040 and fossil fuels are predicted to continue to supply almost 80 percent of world's energy demand towards 2040. As a consequence, global energy-related carbon dioxide emissions are projected to increase by 46% in the same period of time given the current policies and regulations.

Currently, about 95% of all manufactured chemicals originate from fossil resources and only around 5% from renewable resources [44] that in principle are unlimited because they can be replaced over time, and only a fraction of this is from biomass conversion in microbial processes.

Also public and political concern about the volatile global oil market has been raised to advocate for the need to use renewable resources instead. It is a strong argument as far as transportation fuels are concerned, but it is less convincing for the manufacture of biochemicals because less than 5% of the global oil consumption is required to synthesize chemicals. In the US it is around 3% [45].

## 3. Microbial VFA Processes from Biomass

Biological catalysis from renewable feedstocks may be an attractive alternative to petrochemical multistep reactions that are employed in traditional VFA synthesis. The total energy input is generally reduced in biocatalysis compared to petrocatalysis because biocatalysis is occurring at low temperature and low pressure. Moreover, use of heavy metals is abandoned, the use of organic solvents and strong acids and bases is reduced, and fewer by-products are produced by biocatalysis because microbial enzymes are highly specific [46–48]. But downstream processing such as recovery of acids from the fermentation broth is a challenge both technically [49, 50] and economically [51].

**Figure 1:**
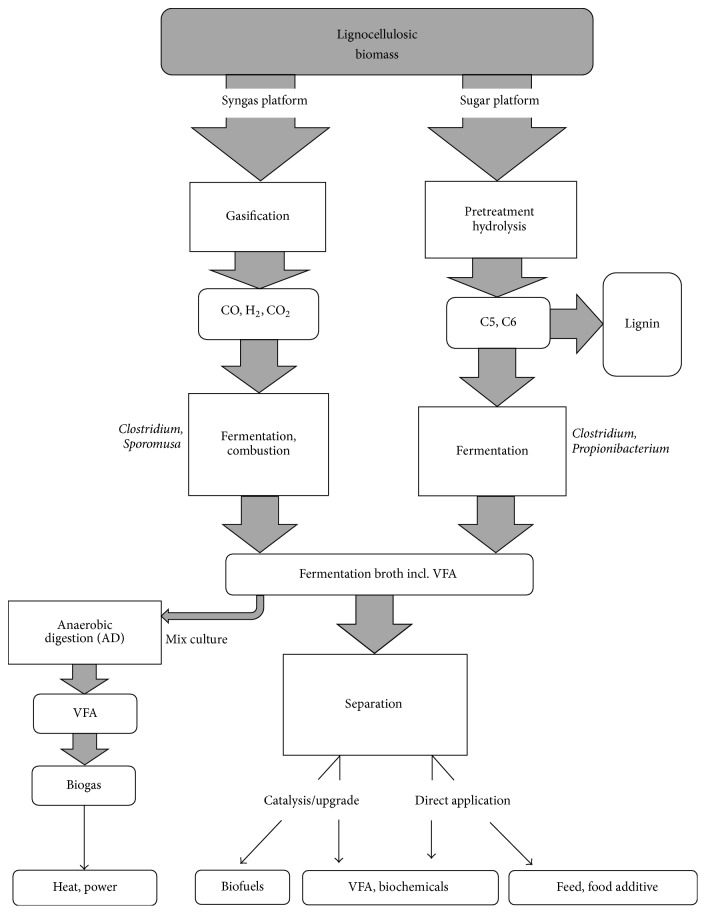
Routes for biomass conversion via one of two platform processes. The sugar platform via pretreatment to microbial fermentation and the syngas platform via gasification to microbial fermentation or combustion. Process feedstock is lignocellulosic biomass and fermentation products are VFA, other biochemicals, and biofuels.

Biomass conversion in a sustainable and biological production of chemicals can proceed by either of two routes in a biorefinery (Figure 1). Feedstock comprising, for example, plant biomass may be processed either via carbohydrate extraction, which constitutes the sugar platform, or via gasification of the biomass, which constitutes the syngas platform. In the first case, the sugar platform is basis for fermentation of C5 and C6 sugars and the produced metabolites such as VFA may be readily used or subjected to subsequent upgrading by chemical catalysis [52]. In the second case, the syngas is converted in gas fermentation predominantly with fuels as principal products [53, 54]. Biomass conversion to intermediate VFAs by anaerobic digestion, which in turn are converted into biogas, is also feasible [55] but is not treated in this review.

Anaerobic VFA production is a result of microbial fermentation where the enzymes convert various carbon substrates to energy (ATP), reducing agents, and intermediates used in anabolic processes and a number of metabolites such as acetic, propionic, and butyric acids. Acetic acid is produced anaerobically through the glycolytic pathway and via pyruvate intermediates as a coproduct by a number of organisms that produce, for example, propionic and butyric acids as their major products.* Propionibacterium* and* Clostridium* are examples of genera capable of these transformations. A conventional fermentation route to acetic acid is via ethanol using, for example,* Acetobacter aceti* [56]. Direct aerobic acetic acid production is conducted by using* Escherichia coli* as biocatalyst in renewable sugar fermentation [57], and* Saccharomyces cerevisiae* has also been used for this purpose [5]. Propionic acid biosynthesis by* Propionibacterium *species takes place via a glycolytic and then a dicarboxylic production pathway through pyruvate and succinate intermediates [58]. Butyric acid is the end product from metabolism of sugars via glycolysis and then through a dicarboxylic production pathway via pyruvate [59].

The split pathways in both propionic and butyric acid fermentations also yield acetic acid (and ATP) that drains the carbon pool, which in turn affects both the final titers and the final recovery of propionic and butyric acids.

The current state of biomass conversion to VFA by microbial processes is still limited. Anaerobic fermentation of plant biomass-derived carbohydrates for direct production of acetic acid is currently not a significant R&D topic, probably because high-value acetic acid is cheaply derived from aerobic conversion of alcohol-containing solutions produced from sugar-containing juice fermentation by acetic acid producers.

The most important organisms used for this process are aerobic bacteria belonging to genera such as* Acetobacter *and* Gluconacetobacter* that produce up to 150 g/L [60]. The food-grade solution derived from this process is vinegar, which is a high-value commodity. Extensive R&D is conducted to improve biological acetic acid production using, for example, acid and thermotolerant strains in aerobic submerged fermentations [61] at different oxygen concentrations [62], with adapted or transformed strains expressing heterologous genes conferring acid tolerance [63], or with genes that promote production capabilities in organisms such as* A. polyoxogenes* that produces around 100 g/L with a rate of 4 g/L/h [64].

Conversion of biomass to acetic acid without the process of microbial fermentation has been investigated [65] and it is well established that biomass degradation using harsh methods leads to formation of significant amounts of acetic acid and other compounds by partial degradation of the plant cell constituents [66]. It is, however, feasible to produce acetic acid from plant biomass by employing mild solvents such as super- and subcritical water instead [67]. For example, increased yields and purity of acetic acid was obtained in an oxidation process conducted at low pressure and without an acid catalyst [68]. The energy demand of such a process is made up of the requirements for oxygen and heating. Lastly, efficient utilization of monoculture microbial conversion of plant biomass by some kind of coproduction [69] of acetic acid and other commercially interesting metabolites has not been reported either through the use of acetic acid bacteria or by using other anaerobic bacteria. However, Brazil based company Braskem has taken out a patent for anaerobic coproduction of acetic acid and isoprene by a genetically modified microorganism in a sustainable process [70].

Propionic acid is the principal fermentation product of bacteria belonging to the* Propionibacterium *genus and propionic acid production from cheese whey substrate, or other lactose effluents, has been studied since 1923. The conclusions from these studies were very often that the slowness of the process was unacceptable for industrial use except in, for example, cheese production [71]. However, propionic acid production from carbohydrate substrates other than lactose is feasible.

Propionic acid production by* Propionibacterium freudenreichii *cells from sugar cane molasses and waste cells was studied in plant fibrous-bed bioreactors (PFB). With nontreated molasses as carbon source, 12.69 g/L of propionic acid was achieved in 120 hours in stirred fermentation, whereas fed-batch fermentation of hydrolyzed molasses in PFB yielded 79.81 g/L of propionic acid within 302 hours fermentation at a rate of 0.26 g/L/h [72] with recycled cells as a nitrogen source.

In fed-batch fermentation with wild-type* P. acidipropionici *cells and corncob molasses containing high concentration of xylose as substrate, the metabolism resulted in a final 71.8 g/L of propionate and a productivity of 0.28 g/L/h [73].

Glycerol is a residual product from biodiesel production and is commonly used in anaerobic fermentations. In two studies [74, 75], improved propionic acid production was obtained by using a metabolically engineered* Propionibacterium jensenii* strain transformed with a plasmid expressing heterologous glycerol dehydrogenase, which is a required enzyme for conversion of glycerol. In a potential-shifted, fed-batch fermentation, a propionic acid concentration of 39.56 g/L was achieved with a productivity of 0.183 g/L/h.

Low-cost feedstocks were used in a study where propionic acid and vitamin B12 were coproduced from hydrolyzed corn stalks, corn steep liquor, and glycerol [76].* P. freudenreichii* subsp.* shermanii* cells were exploited to ferment the mixture, which resulted in 42.7 g/L of propionic acid and a productivity of 0.36 g/L/h. Moreover,* in situ* removal of propionic acid by use of an ion exchange resin during fermentation kept the propionic acid concentration at 10 g/L and resulted in propionic acid concentrations of 91.6 g/L after 258 hours of fermentation yielding 0.71 g/g and a productivity of 0.35 g/L/h.

Propionic acid production was increased by an acid adapted* P. acidipropionici *mutant strain when limiting metabolites were identified and supplemented [77]. These were lactate, fumarate, and succinate metabolites that are known to influence propionic acid synthesis. In a fed-batch fermentation using glycerol as substrate, propionic acid concentrations reached 35 g/L after around 150 hours of fermentation.

The mesophilic* Clostridium tyrobutyricum* has been the preferred organism for R&D in butyric acid production for many years. In a study from 2009 [78], pretreated molasses were used in fed-batch fermentations with adapted and immobilized* C. tyrobutyricum *cells. The cells produced 55.2 g/L of butyric acid and utilized all three available sugars in the molasses (glucose, fructose, and sucrose). The fermentation yield was 0.46 g/g and the productivity was 3.22 g/L/h. Butyric acid production in fermentations with pretreated sugarcane bagasse hydrolysate has also been reported [79]. They constructed a genetically modified strain with an inactivated phosphor transacetylase gene that grew in diluted bagasse hydrolysate. When cells were immobilized in the reactor and the hydrolysate was fed during the fermentation, the cells produced 21 g/L of butyric acid with an average yield of 0.48 g/g and a productivity of 0.51 g/L/h.

Wild-type* C. tyrobutyricum* cells fermented a combination of sweet sorghum stalks and beet molasses in 1 L fed batch and with the cells in suspension [80]. The fermentation resulted in a final butyric acid concentration of 58.8 g/L with a productivity of 1.9 g/L/h and a yield of 0.52 g/g. In a continuous fermentation with* in situ* removal of produced acids by membrane separation [81], a substrate adapted strain was shown to produce butyric acid from a highly concentrated wheat straw hydrolysate as carbon source and urea as nitrogen source. The productivity, yield, and selectivity were 1.30 g/L/h, 0.45 g/g carbohydrates, and 0.88 g/g acids, respectively.

Other scientific approaches, using other* Clostridium *species and other feedstocks than carbohydrates, have been reported in the study of sustainable butyric acid production. For example, a* C. ljungdahlii* strain was transformed to produce butyric acid from carbon dioxide by introducing genes encoding essential enzymes in the butyric acid pathway and by knockout of genes in butyric acid competing pathways such as acetate and ethanol pathways [82]. Up to 70% of the carbon and electron flow in the transformed strain was diverted to production of butyric acid with either H_2_ or CO as electron donors.

VFA production from microbial conversion of lignocellulosic feedstock via the sugar platform is summarized in Table 1. To our knowledge, there are no publicly available reports on microbial VFA production from lignocellulose of propionic acid or VFA production via the alternative syngas platform.

## 4. The Market for Biochemicals in the Bioeconomy

While the pace of innovation of alternative energy technologies has increased markedly during the recent years [92] along with the transition of our energy supply towards a low carbon market [93], we still use hydrocarbons and fossil resource-based chemicals and will probably continue to do so for many years. Industrial biotechnology and related industries will, therefore, become cornerstones in a future bioeconomy in a way with a lower carbon footprint* per capita*.

Industrial biotechnology already exploits the versatility of microbial biosynthesis for the production of many metabolites. The OECD predicts that investments and economic outputs of all types of applied biotechnologies will expand over the coming decades [94]. The main causes for the development of industrial biotechnology in the past were scientific breakthroughs and technological developments, as well as environmental constraints and changes in consumer behavior and demands. For example, the advent, establishment, and growth of modern recombinant DNA technologies have enabled new routes to commercially interesting products via engineered biocatalysts [95–98]. The main causes for driving industrial biotechnology into a future bioeconomy will be somewhat the same as the previous drivers of change, but two additional factors will catalyze the progress and increase the pace. These are the expected growth in the global population [99] that puts constraints on finite natural resources and global climate change.

To address climate change, there is a need to keep a score of the global carbon balance. This will require reducing and replacing the use of fossil resources and over time moving to sustainable raw materials based on residual feedstocks [33, 100], many of which are well suited to biotechnological processing methods. This will demand a development of both green and clean biotechnological processes focusing on efficient conversion of raw materials requiring little input energy and producing a minimum of final waste [98, 101, 102].

The bioeconomy could also create beneficial opportunities for cooperation between sectors that so far have been separated by promoting sustainable development in rural regions having plentiful biomass resources and establishing new linkages between forestry, agricultural, industrial sectors, and universities [103–106] potentially leading to new ways of manufacturing and whole new products [107, 108].

Many stakeholders share interests in obtaining thorough market information concerning chemicals. Such stakeholders include VFA manufacturers, raw material suppliers, end-users of feed, grain and food preservatives, herbicide manufacturers, manufacturers of derivatives and bioplastics, and manufacturing technology providers. But also potential investors in industrial biotechnology for biochemicals require credible market analyses and statistics about consumer demands, market locations, prices, and forecasts. Market data about biochemicals such as VFA are, however, not readily available for the academic world but only from commercial suppliers. Service companies offering market research reports, market analysis, and market forecasts deliver updated reports on demand against a payment of around USD 5000 for access to a 100–300 pages report. In this review we have collected insights and facts about the market for VFA from open sources primarily, but also from commercial suppliers offering limited information free of charge. In Table 2 a number of useful sources available free of charge are presented.

## 5. The Market for Biochemicals

In economic terms such as turnover and the number of employees, the global market for chemicals is significant. According to American Chemistry Council, the global chemical production volume rose by around 10% from 2012 to 2016 [110].

The majority of the chemicals produced are carbon-containing compounds that are supplied from refining of fossil feedstocks. According to a forecast by The European Chemical Industry Council [111], European chemicals industry will remain oil-based over the next decades [111] but, as Cefic points out, there is untapped potential for increased use of biobased feedstocks, not only for the production of specialty chemicals but also of high-volume building block chemicals such as the VFA.

The exact growth rate of the biobased chemicals industry will depend on a number of factors. The relative prices of oil and agricultural raw materials, combined with the speed of technological progress, will be major determinants for switching from fossil to renewable feedstocks.

The US market analysis company MarketsandMarkets foresees that the industry for renewable chemicals will be growing rapidly in the coming years. They estimate that the global market for renewable chemicals will increase from USD 57 billion in 2013 to USD 83.4 billion by 2018, delivering an annual growth rate of 7.7% during the period.

California biotech company Rennovia Inc. is more optimistic regarding relative growth, as they anticipate the global market for renewable chemicals to grow approximately three times during the coming five years! But Rennovia's starting point is a modest USD 3.6 billion of today's market, growing to around USD 12 billion by 2020. The background for such differences is uncertain, but it underlines the need for a critical position and common sense when looking into market forecasts.

Traditional players in the market for chemicals might enter the market for sustainable products by either buying or joining in strategic partnerships with small start-up companies. These start-ups can offer a mature technology platform but have a business plan that lacks, for example, capital or distribution and consumer networks.

Large industrial companies from other sectors that have specific demands for chemicals or technologies may well team up with a biotech company offering exactly this product. For example, a strategic partnership has been established between soft drinks manufacturer The Coca-Cola Company, Austrian ALPLA GmbH that manufactures plastic containers, and the company Avantium to develop a polyethylene furanoate (PEF) recyclable plastic bottle made from plant biomass through their native fermentation-free catalytic synthesis technique YXY technology, which meets the beverage company's requirements and specifications for soft drink bottles.

Although there are prominent examples of biotechnology companies producing chemicals at industrial scale that are derived from plant biomass or other renewable feedstocks, most activity is still at the R&D stage, and this also applies to VFA. Currently, there are a number of short chain fatty acids, which are produced at a larger scale from renewable sources by microbial conversion and many of these are used for polymer plastic applications. Companies featuring established processes and in-house developed technologies are listed in Table 3.

The Dutch company Avantium exploits carbohydrates from plant biomass to produce polyethylene furanoate, a 100% recyclable plastic material featuring improved properties. The carbohydrates are converted to PEF by the company's YXY catalysis technology. They also use the side-streams that are produced when lignocellulosic biomass is pretreated for the recovery of carbohydrates. 5-Hydroxymethylfurfural (HMF) and furfural are two platform chemicals that can be obtained from the dehydration of C6 and C5 sugars. They can be further converted into furanic derivatives such as 2,5-furandicarboxylicacid (FDCA) or furfuryl alcohol (FA) [112], which are precursors to biobased polymers.

British BioSyntha Technology Ltd. is R&D company that develops and sells pilot scale fermentation technology with wild-type or engineered strains. Their fermentation technology produces novel chemicals and novel fuels from gasification of waste plant biomass and other renewable resources.

The New Zealand company LanzaTech produces acetic acid and fuels from microbial conversion of carbon monoxide waste gasses from various sources. Their core technology is the productive microbe used and their separation technology [113]. According to the company they are about to take products from demonstration scale to commercialization and market introduction.

The US company Myriant develops technology for production of lactic and succinic acids. Their business model is based on partnerships and licensing for commercial production: for example, production of lactic acid via fermentation where their Spanish partner Purac has licensed Myriant's process to produce lactic acid and has been producing the chemical on a commercial scale since 2008.

The French company Metabolic Explorer (METEX) produces acids such as butyric acid and alcohols from 2nd-generation biomass. In 2010 the company announced their first industrial pilot phase and validation of PDO and has inaugurated a manufacturing plant in Malaysia. The US based Rennovia Inc. is working on development, scale-up, and commercialization of an array of chemical products from renewable feedstocks coupled with traditional catalysis technology. The company's products are, for example, adipic acid and hexamethylenediamine (HMD), which are building block chemicals of commercial importance.

Verdezyne Inc., a US based company, develops technologies for production of organic acids such as dodecanedioic acid, adipic acid, and sebacic acid with broad applications. In December 2014 the company announced an agreement with Malaysian partner Bio-XCell to construct and run Verdezyne's first commercial-scale renewable manufacturing facility. The product is dodecanedioic acid.

The US based Zeachem Inc. has a demonstration plant facility in Boardman, Oregon, inaugurated in 2012, that produces ethanol and acetic acid from plant biomass feedstock in a hybrid process. Their product capacity for bioacetic acid is currently almost 100,000 L/year. The company has initiated a commercial scale plant to open at the same location with a capacity of nearly 100 million L/year. ZeaChem utilizes a hybrid process consisting of microbial and thermochemical processing in combination, which yields C2 products such as ethanol and acetic acid and derivatives. After pretreatment of the biomass, sugar streams are fermented by homofermentative, thermophilic anaerobes to acetic acid without any microbial CO_2_ production [114]. Acetic acid is then subjected to esterification and the resulting ester is combined with hydrogen to produce ethanol. The hydrogen required to convert the ester to ethanol is derived from syngas produced by gasification of the lignin fraction from the biomass feedstock. The remainder of the syngas is combusted to create steam and power for the process. With process adjustments, the technology can produce three-carbon products including propionic acid, ethyl propionate, propanol, and propylene according the company's own information.

## 6. VFAs Prices and Volumes

Unless clearly stated, all data about prices and volumes supplied in Tables 4, 5, and 6 are based on petrochemical production routes.

## 7. Acetic Acid

According to the market analysis company IHS (2013 figures), a 4-5% growth per year of the global market is expected. Growth will be driven mainly by the Chinese market with a rapid expansion in production facilities and a future consumption growth of acetic acid is expected to be around 7% per year. While the primary application of acetic acid is within the food industry, the second largest global acetic acid use will become production of terephthalic acid (TPA). TPA is mainly used for the manufacture of polyethylene terephthalate (PET) packaging fibers, clothing, plastic bottles, and films. A similar global volume of acetic acid will be used for acetate esters that are exploited mainly as solvents for inks, paints, and coatings.

The US company Celanese and British Petroleum (BP) are among top ranked companies in the world regarding acetic acid production, which is currently produced from oil-derived methanol and carbon monoxide using a chemical catalyst. Therefore, the prospect for growth in sustainable production of acetic acid is dependent upon the nature of feedstocks and processes for methanol production. According to Green Chemicals blog, the total global revenue for biobased production of acetic acid and derivative ethyl acetate amounts to USD 21 billion.

## 8. Propionic Acid

The German company BASF is the largest propionic acid manufacturer in the world and produces 8.8 · 10^4^ tons per year in Germany and China. The acid, which is produced via the petrochemical route, is used in products for feed grain preservation under their trade names Luprosil®, containing propionic acid, and Lupro-Grain®, containing ammonium propionate salt [121]; both products are claimed to reduce CO_2_ emission because drying is not necessary when the grain is conserved. Other major industrial manufacturers, such as US company The Dow Chemical Company, maintain traditional production processes while at the same time developing sustainable production in order to cut production expenses and narrow the gap between fermentation and petrochemical processes [122].

While the market price for propionic acid from the petrochemical route was around 1000 USD/ton in 2012 [119], the price for the acid from the biotechnological route was about 1500–2000 USD/ton. Today propionic acid prices are around 1600–2000 USD/ton while the calcium or sodium salts are slightly cheaper per metric ton. Globally, the use of propionic acid dominates the large market for feed preservatives. The principal use of propionic acid is as an acidifier for animal feed, grain, and food where calcium and sodium propionates accounted for 78.5% of world propionic acid consumption in 2012, according to IHS. Other fast-growing markets include propionate esters such as n-butyl and pentyl propionate because these esters are increasingly being used as replacements for solvents listed as hazardous air pollutants according to IHS. The global market in terms of revenue was estimated to be worth USD 935.7 million in 2012 and is expected to reach USD 1.7 billion by 2018 according to MarketsandMarkets and to grow at a rate of 7.8%–9.6% from 2013 to 2018. While Europe and the US accounted for around two-thirds of the global consumption in 2012, emerging markets such as Asia and Africa are likely to be responsible for future growth in production and use of propionic acid and derivatives.

The major growth in propionic acid demands within a single market niche is the use as an additive to prolong shelf life of preserved food. Private consumers are increasingly demanding “natural” and healthier food additives and propionic acid is “Generally Recognized as Safe” (GRAS) by the US Food and Drug Administration (FDA). Growth in demands for propionic acid is also attributed to the growing demands of organic food products mainly in North America and Western Europe. The trend in natural preservatives originated from European nations wishing to market the clean label food products free of artificial additives especially for preservation of organic foods [123]. An example is the company Danisco's food preservative MicroGard® which is based on propionic acid and marketed as a natural and safe antimicrobial compound.

Thus, changing lifestyles and the fast growth in convenience foods and beverage industry have increased the demand for natural preservatives with expected direct effects on propionic acid demand. By 2016 the global food preservatives market is estimated to reach revenues of USD 2.6 billion, growing at a rate of 2.5% in the coming years thus supporting fast growth amongst “natural” preservatives. Consequently, the future demand for propionic acid is strongly dependent on both food and feed production.

## 9. Butyric Acid

Eastman Chemical Company is a manufacturer of butyric acid via their Oxo Low-Pressure Technology and is one of the major global players in the market. As Eastman foresaw continuous market growth, they expanded their butyric acid production facility in Newport, Tennessee, in 2013/2014 by around 7000 tons/year.

## 10. From Promise to Market

Lignocellulosic biomass can be converted into more than a hundred different chemicals [126]. Among them are new chemicals but also established compounds with immediate drop-in features, such as the VFAs, that can directly substitute for fossil-derived chemicals and constitute platform chemicals, monomers, chemical intermediates, or end products in many industrial sectors.

Because of their functionality (chemical reactivity) and natural origin, the market for acetic, propionic, and butyric acids is already huge today and world market demands for these acids are predicted to grow in the coming years. Although the demand for some chemicals will expire and their use will cease, other chemicals will continue to be in demand, for instance, as platforms and materials but also as liquid transportation fuels. Despite that chemicals and materials can be produced from lignocellulosic biomass by pretreatment and fermentation processes, there are still scientific obstacles in order for biotechnology to become the principal technology for sustainable production of VFAs and other biochemicals within the chemical industry.

The European Technology Platform For Sustainable Chemistry [127] is a joint venture between Cefic and several European chemical organisations that recently published a strategic innovation and research agenda [127], which states that the main obstacle to the spread of biobased chemicals is the supply of sufficient amounts of biomass that are price competitive, and to ensure a stable supply of 2nd-generation biomass, which does not compete with food or feed production. SusChem highlight three areas, which they consider major R&D topics in the development of biobased chemicals: (1) fractionation of biomass into its components and improved pretreatment methods for lignin conversion and (2) development of robust industrial microbial fermentation strains with tailored capabilities such as improved resistance to their own metabolic products and (3) process developments, which combine green chemistry and biotechnology technologies for improvement of biomass utilization and improved economics.

But besides the technical challenges, there are also economic challenges and issues about expenditure and costs are without any doubt of great importance to facilitate transition from an oil-based to a biobased economy. It will require a deliberate and sustained focus on biomass valorization, microbial productivity, and improved processes to reduce total costs of biological VFA production. Costs reduction is feasible: for example, production costs of biosuccinic acid were reduced to 25% over twelve years by keeping economics in focus [128].

As shown in this review, biobased products and materials are structurally identical to those obtained from fossil-based feedstocks and there is a potential to develop new biobased products and materials that cannot be produced from fossil feedstocks. To become truly competitive, biochemicals and biomaterials should, however, be genuinely new or feature improved properties and economics compared to fossil-based products [129].

There are without doubt still technical challenges to be solved [130] before a full scale commercialization of microbial processes for production of renewable chemicals has been marketed and a wide range of biomass-derived biochemicals are available on the global market. On the other hand, the outlook for biotechnology is promising because there has never been a period in the history of biotechnology where interests [131] and needs have been more obvious than presently.

## Figures and Tables

**Table 1:** Microbial VFA production from lignocellulosic biomass.

Classification	VFA	Biomass	Conditions	Productivity	Reference
*C. cellulolyticum*	A.A.	Rice straw	Sugar platform	*Y*: 0.23 g/g	[83]
*C. tyrobutyricum*	B.A.	Rice straw	Sugar platform detoxification	*T*: 8.7 g/L	[84]
*C. tyrobutyricum*	B.A.	Wheat straw Switch grass	Sugar platform pH control	*Y*: 0.44 g/g *Y*: 0.42 g/g	[85]
*C. tyrobutyricum*	B.A.	Wheat straw	Sugar platform acid removal urea supplement	*R*: 1.30 g/L/h *Y*: 0.45 g/g *S*: 0.88 g/g	[81, 86]
*C. tyrobutyricum*	B.A.	Corn fibre	Steep liquor suppl.	*R*: 2.91 g/L/h *Y*: 0.47 g/g	[87]
*C. tyrobutyricum*	B.A.	Rice straw	Sugar platform detoxification	*T*: 8.1 g/L	[88]
*C. thermobutyricum*	B.A.	Sorghum bagasse	Sugar platform 50°C	*T*: 17.6 g/L *Y*: 0.44 g/g	[89]
Mixed culture	B.A.	Rice straw	Sugar platform pH buffered	*Y*: 0.38 g/g *S*: 0.70 g/g	[90]
*T. fusca*	B.A.	Corn stover	Cellulolytic activity aerobic fermentation 55°C	*T*: 2.37 g/L *Y*: 0.52 g/g	[91]

A.A. denotes acetic acid; B.A. denotes butyric acid. *R*: denotes rate; *S*: denotes selectivity; *T*: denotes titer; *Y*: denotes yield.

**Table 2:** Market information sources.

Source	References	Location
The Essential Chemical Industry	http://www.essentialchemicalindustry.org/	York, U.K.
MarketsandMarkets	http://www.marketsandmarkets.com/	Dallas, US
Cefic	http://www.cefic.org/	Brussels, Belgium
Reed Business Information Limited. Reed Elsevier	http://www.icis.com/	London, UK Amsterdam, NL
TD The Market Publishers, Ltd.	https://marketpublishers.com/	Limassol, Cyprus
IHS, Inc. [109]	https://www.ihs.com/ http://www.chemweek.com/	Douglas County, US
Biofuels Digest	http://www.biofuelsdigest.com/	
Green Chemicals Blog	http://greenchemicalsblog.com/	NYC and London
Biotechnology Industry Organisation	https://www.bio.org/	Washington DC, US
Focus on Catalysts	http://www.journals.elsevier.com/focus-on-catalysts	
Biomass Magazine	http://www.biomassmagazine.com/	Grand Forks, US
iBIB2014/15	http://nova-institut.de/	Hürth, Germany

**Table 3:** Biotech companies producing organic acids mainly from fermentation of renewable feedstocks.

Company, reference	Products	Technology platform
Avantium, N.L https://www.avantium.com/	Acids and fuels PEF plastic	Lignocellulosic feedstocks YXY catalysis
BioAmber, Canada https://www.bio-amber.com/	Succinic acid	Fermentation of corn syrup
BioSyntha Technology Ltd., UK http://www.biosyntha.com/	Novel acids and fuels	Plant biomass feedstock syngas fermentation with engineered strains
Cargill, USA http://www.cargillfoods.com/	Citric acid	Carbohydrate fermentation
Genomatica, USA http://www.genomatica.com/	Adipic acid	Sugar and syngas fermentation
LanzaTech, NZ http://www.lanzatech.com/	Acetic acid fuels	Waste gasses feedstocks fermentation hybrid separation
Metabolic Explorer, France http://www.metabolic-explorer.com/	Butyric acid PDO MPG methionine	Second-generation biomass feedstocks and fermentation
Myriant Corporation, USA http://www.myriant.com/	Lactic acid succinic acid	Plant biomass feedstock fermentation
NatureWorks, USA http://www.natureworksllc.com/	Lactic acid	Carbohydrate fermentation
Rennovia Inc., USA http://www.rennovia.com/	Adipic acid hexamethylenediamine	Plant biomass feedstock chemical catalysis
Reverdia, Netherlands http://www.reverdia.com/	Succinic acid	Yeast fermentation of starch
Succinity, Germany http://www.succinity.com/	Succinic acid	Bacterial fermentation of biomass
Verdezyne Inc., USA http://www.verdezyne.com/	Dodecanedioic acid adipic acid sebacic acid	Plant biomass feedstock fermentation using engineered yeast
Zeachem Inc., USA http://www.zeachem.com/	Acetic acid ethanol ethyl acetate	Plant biomass feedstock fermentation

**Table 4:** Global market and prices of acetic acid.

Year	Production^1^ (t)	Price^2^ (USD/t)	Reference
2000	8.3 · 10^6^		[22]
2008	1 · 10^7^		[115]
2014	1.5 · 10^7^		http://www.essentialchemicalindustry.org/
2014		500–850	http://www.alibaba.com/
2015	1.6 · 10^7^		http://www.lanzatech.com/

^1^Data are supplied either as actual production from petrochemistry or as production capacity.

^2^Price is purity and quantity dependent. Free On Board (FOB).

**Table 5:** Global market and prices of propionic acid.

Year	Production^3^ (t)	Price^4^ (USD/t)	Reference
1992	1 · 10^5^		[116]
1996	1.8 · 10^5^		[22]
1997	1.9 · 10^5^		[22]
1999	2 · 10^5^		[22]
2006	1.3 · 10^5^ ^5^ 3.5 · 10^5^ 3.8 · 10^5^		[117] http://www.marketsandmarkets.com/ [118]
2012		1000	[119]
2014	3.8 · 10^5^	1500–2000 1600–2300	[120] http://www.lookchem.com/ http://www.alibaba.com/

^3^Data are supplied either as actual production from petrochemistry or as production capacity.

^4^Price is quality and quantity dependent. Free On Board (FOB).

^5^In 2006, a minor fraction was produced by fermentation and commercialized for food and fragrance manufacturing [117].

**Table 6:** Global market and prices for butyric acid. In 2006, a minor fraction was produced by fermentation and commercialized for food and fragrance manufacturing [117].

Year	Production^6^ (t)	Price^7^ (USD/t)	Reference
2008	5 · 10^4^		[124]
2011	5 · 10^5^		[125]
2014		1800–1900	http://www.alibaba.com/

^6^Data are supplied either as actual production from petrochemistry or as production capacity.

^7^Price is quality and quantity dependent. Free On Board (FOB).
